# Exosomal microRNA profiling to identify hypoxia-related biomarkers in prostate cancer

**DOI:** 10.18632/oncotarget.24532

**Published:** 2018-02-17

**Authors:** Gati K. Panigrahi, Anand Ramteke, Diane Birks, Hamdy E. Abouzeid Ali, Sujatha Venkataraman, Chapla Agarwal, Rajeev Vibhakar, Lance D. Miller, Rajesh Agarwal, Zakaria Y. Abd Elmageed, Gagan Deep

**Affiliations:** ^1^ Department of Cancer Biology, Wake Forest School of Medicine, Winston-Salem, North Carolina, USA; ^2^ Department of Molecular Biology and Biotechnology, Tezpur University, Tezpur, Assam, India; ^3^ University of Colorado Denver, Aurora, Colorado, USA; ^4^ Department of Pharmaceutical Sciences, Texas A&M Rangel College of Pharmacy, Kingsville, Texas, USA; ^5^ Wake Forest Baptist Comprehensive Cancer Center, Winston-Salem, North Carolina, USA; ^6^ Department of Urology, Wake Forest School of Medicine, Winston-Salem, North Carolina, USA

**Keywords:** prostate cancer, hypoxia, exosomes, miRNAs, biomarkers

## Abstract

Hypoxia and expression of hypoxia-related biomarkers are associated with disease progression and treatment failure in prostate cancer (PCa). We have reported that exosomes (nanovesicles of 30-150 nm in diameter) secreted by human PCa cells under hypoxia promote invasiveness and stemness in naïve PCa cells. Here, we identified the unique microRNAs (miRNAs) loaded in exosomes secreted by PCa cells under hypoxia. Using TaqMan^®^ array microRNA cards, we analyzed the miRNA profile in exosomes secreted by human PCa LNCaP cells under hypoxic (Exo^Hypoxic^) and normoxic (Exo^Normoxic^) conditions. We identified 292 miRNAs loaded in both Exo^Hypoxic^ and Exo^Normoxic^. The top 11 miRNAs with significantly higher level in Exo^Hypoxic^ compared to Exo^Normoxic^ were miR-517a, miR-204, miR-885, miR-143, miR-335, miR-127, miR-542, miR-433, miR-451, miR-92a and miR-181a; and top nine miRNA with significantly lower expression level in Exo^Hypoxic^ compared to Exo^Normoxic^ were miR-521, miR-27a, miR-324, miR-579, miR-502, miR-222, miR-135b, miR-146a and miR-491. Importantly, the two differentially expressed miRNAs miR-885 (increased expression) and miR-521 (decreased expression) showed similar expression pattern in exosomes isolated from the serum of PCa patients compared to healthy individuals. Additionally, miR-204 and miR-222 displayed correlated expression patterns in prostate tumors (Pearson *R* = 0.66, *p* < 0.0001) by The Cancer Genome Atlas (TCGA) prostate adenocarcinoma (PRAD) genomic dataset analysis. Overall, the present study identified unique miRNAs with differential expression in exosomes secreted from hypoxic PCa cells and suggests their potential usefulness as a biomarker of hypoxia in PCa patients.

## INTRODUCTION

Prostate cancer (PCa) is the most common non-cutaneous cancer among men worldwide and a leading cause of cancer-related death in the United States and in Europe [1]. An estimated 180,890 men were diagnosed with PCa in the United States in 2016, and nearly 26,120 men died from it [1]. The diagnostic use of prostate-specific antigen (PSA) has resulted in early diagnosis, and has substantially increased the number of new PCa cases. However, there is no consensus regarding whether PSA screening effectively reduces the risk of death from the disease. The poor association between disease state and PSA levels sometimes leads to needless diagnoses and overtreatment of indolent PCa [2]. Because of the molecular heterogeneity of PCa [3], identification and testing of molecular markers may be a rational approach to expedite PCa diagnosis, prognosis, and treatment. Apart from proteins and messenger RNAs (mRNAs), which have shown utility in several clinical scenarios, there is growing interest regarding the utility of microRNAs (miRNAs) as PCa biomarkers. miRNAs are a group of small (<22 nt) noncoding RNAs that regulate gene expression post-transcriptionally by targeting their corresponding mRNAs. These miRNAs can be isolated from a variety of biological samples [4, 5]. Additionally, circulating cell-free miRNAs are extremely resistant to ribonucleases, extreme pH conditions, and freeze-thawing because of their packaging in microvesicles or exosomes [6].

Exosomes are an intriguing class of nano-sized (30–150 nm in diameter) extracellular vesicles secreted by cells for intercellular communication. Most cell types, including cancer cells, secrete exosomes in body fluids, including blood, urine, semen, milk and saliva. They carry a wealth of information about disease characteristics in their unique cargo proteins, lipids, nucleic acids, and metabolites [7] which are unique to specific diseases and types of cells [8]. Biomarker mining from exosomes has emerged as an attractive opportunity mainly because various components are well protected within the lipid bilayer and preserved for longer duration without any significant loss of information [9]. Furthermore, compared to complex plasma samples, exosomes provide a much cleaner clinical sample, thus easing analyses [7]. Hence, analysis of exosomes is an avenue for developing novel protein and nucleotide biomarkers for cancer diagnosis and prognosis. For example, McKiernan *et al.* identified a noninvasive 3-gene expression assay in urinary exosomes that can discriminate clinically relevant Gleason-grade 7 diseases from low-grade (Gleason-grade 6) PCa [10]. This is a huge advancement as currently overdiagnosis and unnecessary PCa treatment are immense clinical problems.

The tumor microenvironment plays a crucial role in tumor growth, angiogenesis, epithelial-to-mesenchymal transition (EMT) and metastasis [11]. Intercellular communication between tumor and tumor microenvironment components is mediated by paracrine signaling involving growth factors, chemokines, and proteinases [11–13]. Recent reports have suggested the contribution of exosomes and microvesicles in intercellular communication in the local tumor microenvironment and in preparation of pre-metastatic niches in secondary organs [14, 15]. Hypoxia (low oxygen conditions) in prostate tumors is associated with an aggressive phenotype and poor prognosis [16] and induces genetic and proteomic changes in cancer cells by selecting for clones with enhanced invasiveness, drug resistance, and stemness [17]. Therefore, a non-invasive biomarker to assess hypoxia status in PCa is warranted to better understand the pathogenesis of PCa.

Recently, we reported that exosomes secreted by PCa cells under hypoxic conditions (Exo^Hypoxic^) promote motility, invasiveness and stemness of naive PCa cells and promote cancer-associated fibroblasts (CAF)-type phenotype in prostate fibroblasts [18]. We also identified that Exo^Hypoxic^ are loaded with higher amount and number of proteins. We then identified the lipid signature in hypoxic PCa cells and their Exo^Hypoxic^, which may serve as a biomarker to assess the aggressiveness of malignant tumors [19]. In the present investigation, we aimed to profile the exosomal miRNAs in PCa cells (LNCaP) both under hypoxic and normoxic conditions. This study identified a unique set of miRNAs loaded in hypoxic PCa exosomes that could contribute to enhanced invasiveness, stemness, and microenvironmental changes [18, 19] and might be of clinical use as non-invasive blood-based biomarkers to assess hypoxia in primary tumors.

## RESULTS

### Expression profile of miRNA in Exo^Normoxic^ and Exo^Hypoxic^ secreted by PCa cells.

Exosomes were isolated from human PCa LNCaP cells cultured under hypoxic (1% O_2_, Exo^Hypoxic^) and normoxic (∼21% O_2_, Exo^Normoxic^) conditions. Thereafter, miRNAs were isolated from Exo^Normoxic^ and Exo^Hypoxic^ and profiled by TaqMan^®^ Array MicroRNA Cards with a pre-amplification kit. Out of a total of 384 unique miRNAs, 292 were loaded in both Exo^Hypoxic^ and Exo^Normoxic^ (Supplementary Table 1); the remaining 92 miRNAs has fewer than 2 successful measurements in at least 1 condition. Approximately 56 of the 92 miRNAs were not detected in any samples. The top 20 miRNAs significantly up- or down-regulated in Exo^Hypoxic^ compared to Exo^Normoxic^ are listed in Table 1. Nine miRNAs were expressed at a significantly lower level in Exo^Hypoxic^ compared to Exo^Normoxic^: miR-521 (Fold change = 0.0005), miR-27a (Fold change = 0.24), miR-324 (Fold change = 0.446), miR-579 (Fold change = 0.448), miR-502 (Fold change = 0.396), miR-222 (Fold change = 0.232), miR-135b (Fold change = 0.325), miR-146a (Fold change = 0.456) and miR-491(Fold change = 0.482). On the other hand, 11 miRNAs were significantly higher in Exo^Hypoxic^ compared to Exo^Normoxic^: miR-517a (Fold change = 7.18), miR-204 (Fold change = 6.62), miR-885 (Fold change = 2.62), miR-143 (Fold change = 2.55), miR-335 (Fold change = 2.06), miR-127 (Fold change = 2.49), miR-542 (Fold change = 4.32), miR-433 (Fold change = 3.13), miR-451 (Fold change = 1.87), miR-92a (Fold change = 1.65) and miR-181a (Fold change = 1.51).

**Table 1:** miRNAs Significantly Up- and Downregulated in Exo^Normoxic^ versus Exo^Hypoxic^

Sl No	Target Name	Normoxia range	Hypoxia range	*p* Value	Fold Change	Change under hypoxia
1	hsa-miR-521-001122	0.3820	0.4270	0.0007	0.0005	DOWN
2	hsa-miR-27a-000408	0.8950	0.9850	0.0066	0.2468	DOWN
3	hsa-miR-324-3p-002161	0.7490	0.4330	0.0104	0.4462	DOWN
4	hsa-miR-579-002398	0.6610	0.8510	0.0215	0.4485	DOWN
5	hsa-miR-502-001109	0.6150	1.0410	0.0225	0.3963	DOWN
6	hsa-miR-222-002276	1.5440	1.4600	0.0304	0.2325	DOWN
7	hsa-miR-135b-002261	1.4240	0.8850	0.0395	0.3259	DOWN
8	hsa-miR-146a-000468	0.9840	0.8200	0.0401	0.4562	DOWN
9	hsa-miR-491-5p-001630	1.1460	0.5680	0.0484	0.4822	DOWN
10	hsa-miR-517a-002402	1.2900	1.7650	0.0124	7.1834	UP
11	hsa-miR-204-000508	1.0390	2.0000	0.0484	6.6246	UP
12	hsa-miR-885-5p-002296	1.1280	0.4070	0.0159	2.6250	UP
13	hsa-miR-143-002249	0.3210	1.0210	0.0182	2.5568	UP
14	hsa-miR-335-000546	0.8500	0.4390	0.0210	2.0634	UP
15	hsa-miR-127-000452	0.6730	1.0910	0.0316	2.4932	UP
16	hsa-miR-542-5p-002240	0.2180	0.8120	0.0374	4.3229	UP
17	hsa-miR-433-001028	1.4870	1.1720	0.0411	3.1383	UP
18	hsa-miR-451-001141	0.3140	0.3020	0.0030	1.8738	UP
19	hsa-miR-92a-000431	0.5130	0.4160	0.0191	1.6548	UP
20	hsa-miR-181a-000480	0.2140	0.5160	0.0275	1.5161	UP

### Ingenuity pathway analysis (IPA) and prediction of miRNA targets

To identify possible functions of expressed miRNAs in Exo^Normoxic^ and Exo^Hypoxic^, we predicted miRNA targets using the microRNA Target Filter function in IPA software. All 20 significantly modulated miRNAs in Exo^Normoxic^ and Exo^Hypoxic^ were analyzed for target mRNA identification. Among these, 15 miRNAs (miR-143, miR-146a, miR-181a, miR-204, miR-222, miR-27a, miR-335, miR-433, miR-491, miR-502, miR-521, miR-92a, miR-127, miR-135b and miR-451) target 211 mRNAs when the confidence limit was set as “Experimentally Observed” only. The miRNA-mRNA pairs and source are listed in Table 2.

**Table 2:** Target mRNAs regulated by corresponding miRNAs, analyzed by IPA

ID	Source	Targeted mRNA (Symbol)
hsa-miR-127	TarBase, miRecords	BCL6, PRDM, RTL1, XBP1
hsa-miR-135b	Ingenuity Expert Findings, TargetScan Human, miRecords	ALOX5AP, APC, HTR1A, JAK2, RUNX2, SLC6A4, SMAD5, TRPS1
hsa-miR-143	Ingenuity Expert, Findings, TarBase, TargetScan Human, miRecords	BCL2, DNMT3A, FNDC3B, IGFBP5, KRAS, MAPK12, MAPK7, MDM2, PLK1, PRC1, TOP2A
hsa-miR-146a	Ingenuity Expert Findings,TargetScan Human,miRecords	ATOH8, BLMH, BRCA1, C8A, CAMP, CCL8, CCNA2, CCR3, CD1D, CD40, CDKN3, CFH, CHUK, COL13A1, CRP, CXCL8, CXCR4, DMBT1, FADD, IFNA1/IFNA13, IFNB1, IL10, IL12RB2, IL1F10, IL1R1, IL1RAP, IL1RAPL2, IL1RL2, IL36A, IL36B, IL36G, IL36RN, IL37, IRAK1, IRAK2, IRF5, KIF22, LALBA, LBP, LTB, LTF, MCM10, MCPH1, METTL7A, MMP16, MR1, NFIX, NLGN1, NOS2, NOVA1, PA2G4, PBLD, PDIK1L, PEX11G, PGLYRP1, PGLYRP2, PLEKHA4, POLE2, PRR15, PTAFR, PTGES2, RAD54L, S100A12, SDCBP2, SFTPD, STAT1, SYT1, TIMELESS, TLR1, TLR10, TLR4, TLR9, TMSB15A, TRAF6, TRIM14, UHRF1, VWCE
hsa-miR-181a	Ingenuity Expert Findings,TarBase,TargetScan Human,miRecords	AICDA, BCL2, CD69, CDKN1B, CDX2, ESR1, GATA6, GRIA2, HOXA11, KRAS, NLK, NOTCH4, PLAG1, TCL1A, TIMP3, TRA, VSNL1
hsa-miR-204	Ingenuity Expert Findings,TargetScan Human, miRecords	ARPC1B, ATP2B1, AURKB, BMP1, CDC25B, CDH11, CTSC, EFNB1, ERF, FBN2, HMGA2, HOXB7, ITGB4, MMP3, MMP9, SHC1, SOX4, SPARC, SPDEF, TRPS1
hsa-miR-222	Ingenuity Expert Findings,TarBase,TargetScan Human,miRecords	BBC3, BCL2L11, BMF, BNIP3L, CDKN1B, CDKN1C, DDIT4, DIRAS3, ESR1, FOS, FOXO3, ICAM1, KIT, MMP1, PIK3R1, PPP2R2A, PTEN, PTPRM, SOD2, TBK1, TIMP3
hsa-miR-27a	Ingenuity Expert Findings,TargetScan Human,miRecords, TarBase	ADORA2B, BAX, BBC3, CTNNBIP1, CYP1B1, FADD, FBXW7, FOXO1, GCA, GRB2, IFI16, IGF1, MEF2C, MMP13, NOTCH1, ODC1, PDPK1, PEX7, PHB, PKMYT1, PMAIP1, PPARG, PXN, RUNX1, RXRA, SMAD3, SMAD4, SMAD5, SRM, ST14, THRB, WEE1, ZBTB10
hsa-miR-335	Ingenuity Expert Findings,TargetScan Human	KIT, PTPN11, PXN, RASA1, SRF
hsa-miR-433	TarBase, miRecords	FGF20, HDAC6, RTL1
hsa-miR-451	Ingenuity Expert Findings, TargetScan Human, miRecords	ABCB1, AKTIP, FBXO33, MIF
hsa-miR-491-5p	Ingenuity Expert Findings,TargetScan Human	BCL2L1, GIT1
hsa-miR-502	TargetScan Human, miRecords	KMT5A
hsa-miR-521	Ingenuity Expert Findings	ERCC8
hsa-miR-92a	Ingenuity Expert Findings,TargetScan Human,miRecords	BCL2L11, BMPR2, CCNE2, CDKN1A, CDKN1C, ENPP6, FBXW7, HIPK3, IKZF1, ITGA5, ITGB3, MAP2K4, MAPRE1, MYLIP, OSBPL2, OSBPL8, PCGF1, PTEN, RFFL, VSNL1, ZEB2,

Next, we performed core analysis of the above 211 mRNAs using IPA software to predict involvement of different pathways. Target mRNAs were grouped into top canonical pathways, diseases and Biological functions (e.g. Diseases and disorders, Molecular and cellular functions, Physiological system development and functions) and Toxicologic functions (e.g. Assays: Clinical chemistry and hematology, Cardiotoxicity, Hepatotoxicity and Nephrotoxicity). Results are summarized in Table 3. The canonical pathways (with –log (*p*-value) more than 15) are depicted in Figure 1. The top five canonical pathways for genes that are targets of differentially expressed microRNAs were IL-6 signaling (*p* value: 6.46E-28), Role of Osteoblasts, Osteoclasts and Chondrocytes in Rheumatoid Arthritis (*p* value: 1.22E-26), Role of Macrophages, Fibroblasts and Endothelial Cells in Rheumatoid Arthritis (*p* value: 4.48E-26), Molecular Mechanisms of Cancer (*p* value: 3.75E-24), and Acute Phase Response Signaling (*p* value: 7.59E-21).

**Table 3:** Analysis of 211 target mRNAs by ingenuity pathway analysis

Molecular and Cellular Functions	*p*-value
Cellular Movement	5.12E-13–3.33E-49
Cell Death and Survival	5.91E-13–3.60E-49
Cellular Development	1.06E-13–1.07E-48
Cellular Growth and Proliferation	9.86E-14–1.56E-44
Cell-To-Cell Signaling and Interaction	5.92E-13–2.43E-38
**Diseases and Disorders**	***p*-value**
Inflammatory Response	6.90E-13–9.60E-39
Cancer	6.83E-13–1.73E-34
Organismal Injury and Abnormalities	7.48E-13–1.73E-34
Connective Tissue Disorders	4.78E-14–1.60E-29
Inflammatory Disease	6.87E-14–1.60E-29
**Physiological System Development and Function**	***p*-value**
Organismal Survival	9.57E-42–3.36E-47
Hematological System Development and Function	6.90E-13–3.62E-41
Tissue Morphology	6.90E-13–3.62E-41
Immune Cell Trafficking	3.61E-13–1.27E-38
Hematopoiesis	1.06E-13–2.83E-35
**Top Canonical Pathways**	***p*-value**
IL-6 Signaling	6.46E-28
Role of Osteoblasts, Osteoclasts and Chondrocytes in Rheumatoid Arthritis	1.22E-26
Role of Macrophages, Fibroblasts and Endothelial Cells in Rheumatoid Arthritis	4.48E-26
Molecular Mechanisms of Cancer	3.75E-24
Acute Phase Response Signaling	7.59E-21
**Top Tox Functions: Assays-Clinical Chemistry and Hematology**	***p*-value**
Increased Levels of Alkaline Phosphatase	3.45E-08–3.45E-08
Increased Levels of Red Blood Cells	8.43E-06–8.43E-06
Increased Levels of ALT	5.90E-02–4.28E-05
Increased Levels of Creatinine	4.89E-05–4.89E-05
Increased Levels of AST	9.73E-03–9.73E-03
**Top Tox Functions: Cardiotoxicity**	***p*-value**
Cardiac Necrosis/Cell Death	2.05E-03–7.99E-15
Cardiac Hypertrophy	5.78E-02–1.76E-14
Heart Failure	2.99E-01–1.23E-08
Cardiac Infarction	3.97E-02–1.32E-08
Congenital Heart Anomaly	8.71E-02–3.74E-08
**Top Tox Functions: Hepatotoxicity**	***p*-value**
Liver Necrosis/Cell Death	1.67E-01–2.68E-27
Hepatocellular Carcinoma	1.14E-01–3.69E-12
Liver Hyperplasia/Hyperproliferation	1.32E-01–3.69E-12
Liver Damage	6.85E-02–1.26E-11
Liver Proliferation	1.67E-01–6.30E-11
**Top Tox Functions: Nephrotoxicity**	***p*-value**
Renal Inflammation	5.00E-01–1.65E-12
Renal Nephritis	5.00E-01–1.65E-12
Renal Necrosis/Cell Death	2.00E-01–1.57E-11
Renal Proliferation	5.90E-02–9.32E-07
Glomerular Injury	1.14E-01–1.82E-06

**Figure 1:**
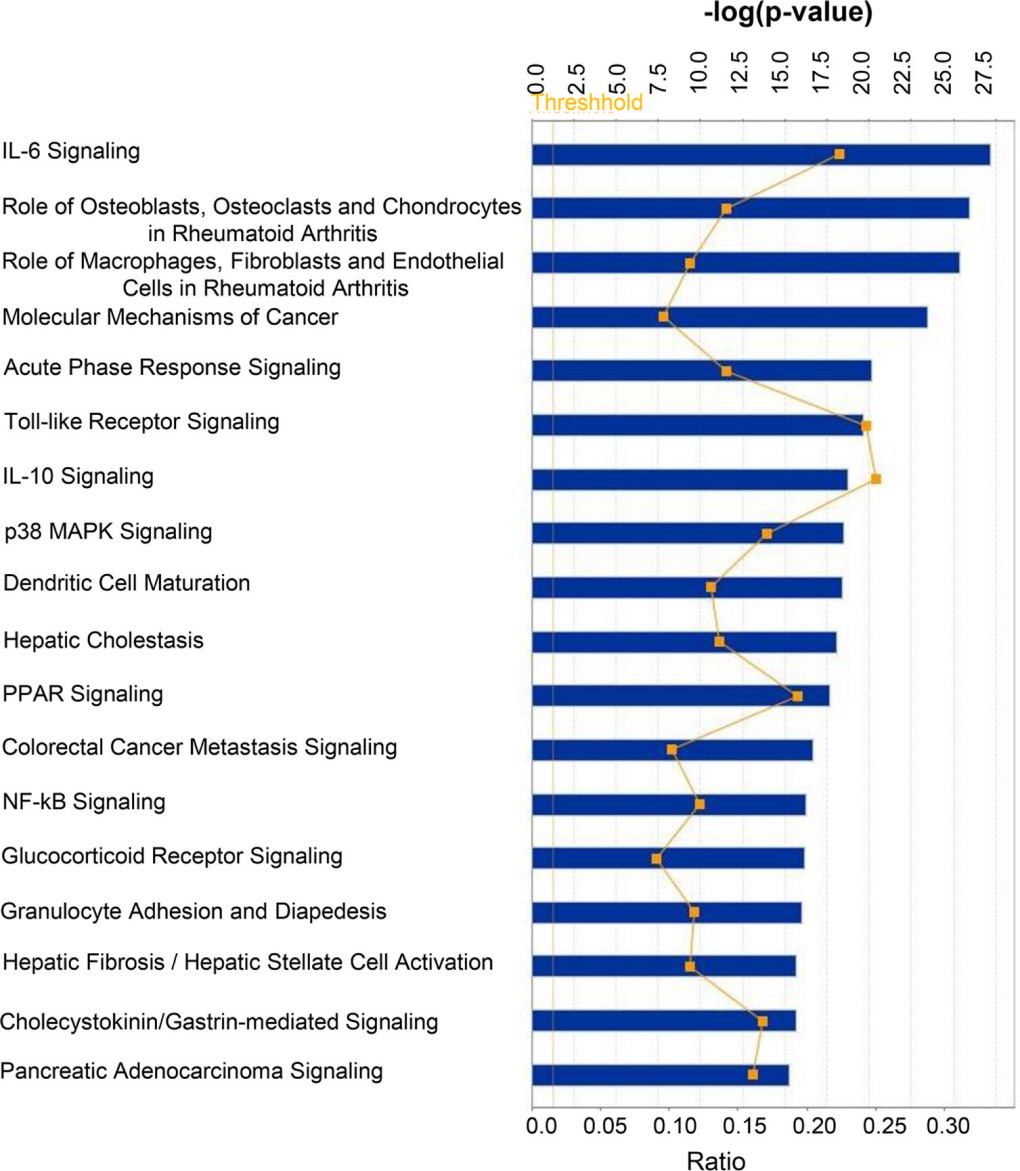
Top canonical pathways targeted by mRNAs whose expression is regulated by miRNAs differentially loaded in Exo^Hypoxic^ from human PCa LNCaP cells Data were analyzed by IPA; pathways having –log (*p*-value) more than 15 are presented with threshold value 0.5. Fisher’s exact test was used to calculate a *P*-value determining the probability of the association between the genes and the dataset in the canonical pathways.

**Figure 2:**
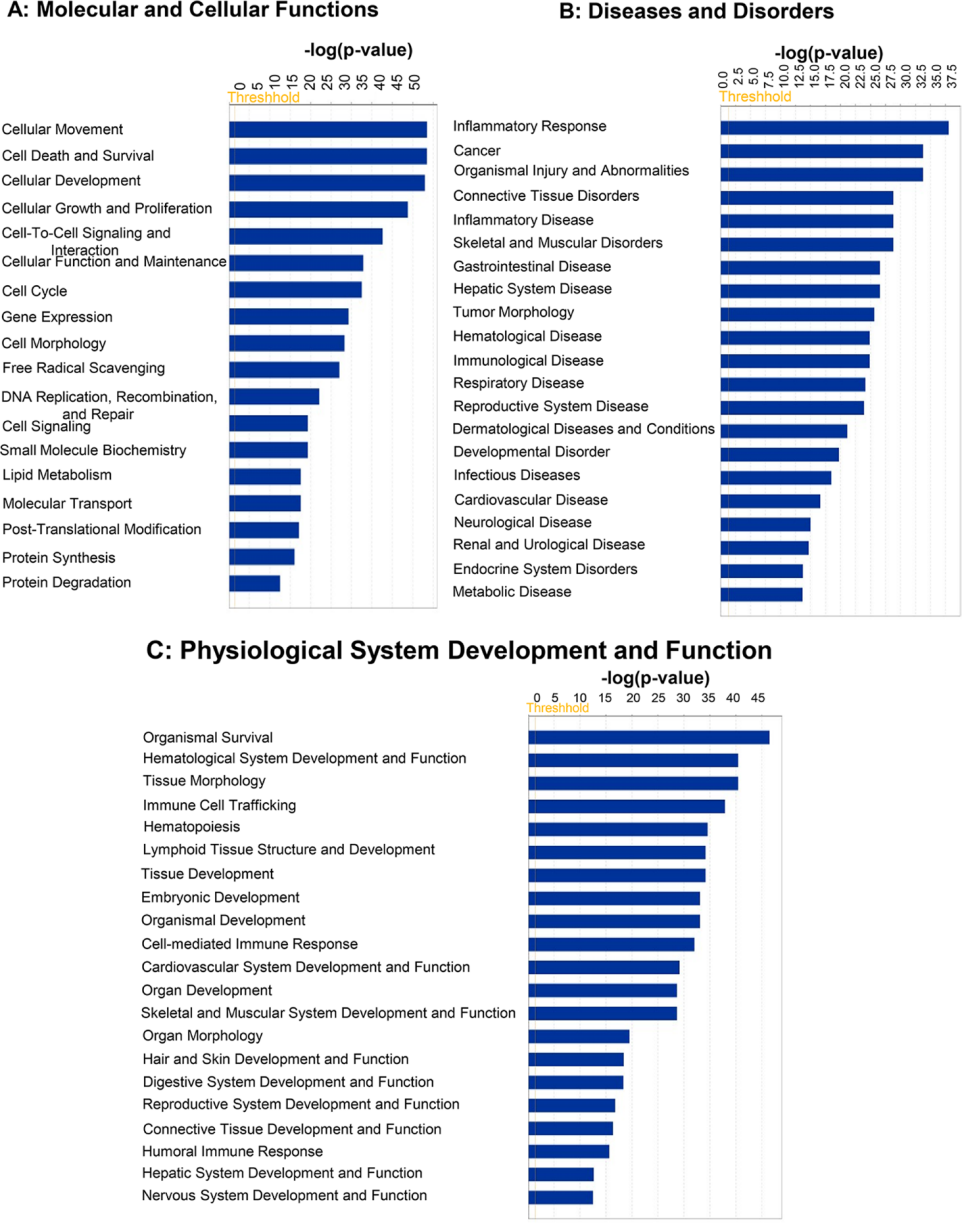
Top predicted “Disease and Bio functions” targeted by mRNAs whose expression is regulated by miRNAs differentially loaded in Exo^Hypoxic^ from human PCa LNCaP cells Data were analyzed by IPA. Top (**A**) ‘Molecular and Cellular Functions’; (**B**) ‘Disease and Disorders’; and (**C**) ‘Physiological System Development and Function’ are presented. Fisher’s exact test was used to calculate a *P*-value determining the probability of the association between the genes and the dataset in the canonical pathways.

The top five Molecular and Cellular Functions were Cellular Movement (*p* value: 5.12E-13–3.33E-49), Cell Death and Survival (*p* value: 5.91E-13–3.60E-49), Cellular Development (*p* value: 1.06E-13–1.07E-48), Cellular Growth and Proliferation (*p* value: 9.86E-14–1.56E-44), and Cell-To-Cell Signaling and Interaction (*p* value: 5.92E-13–2.43E-38) (Figure 2A). The top five Diseases and Disorders were Inflammatory Response (*p* value: 6.90E-13–9.60E-39), Cancer (*p* value: 6.83E-13–1.73E-34), Organismal Injury and Abnormalities (*p* value: 7.48E-13–1.73E-34), Connective Tissue Disorders (*p* value: 4.78E-14–1.60E-29), and Inflammatory Disease (*p* value: 6.87E-14–1.60E-29) (Figure 2B). The top five Physiological system development and functions included Organismal Survival (*p* value: 9.57E-42–3.36E-47), Hematological System Development and Function (*p* value: 6.90E-13–3.62E-41), Tissue Morphology (*p* value: 6.90E-13–3.62E-41), Immune Cell Trafficking (*p* value: 3.61E-13–1.27E-38), and Hematopoiesis (*p* value: 1.06E-13–2.83E-35) (Figure 2C).

### Analysis of Top Tox Functions in IPA (Figure 3)

**Figure 3:**
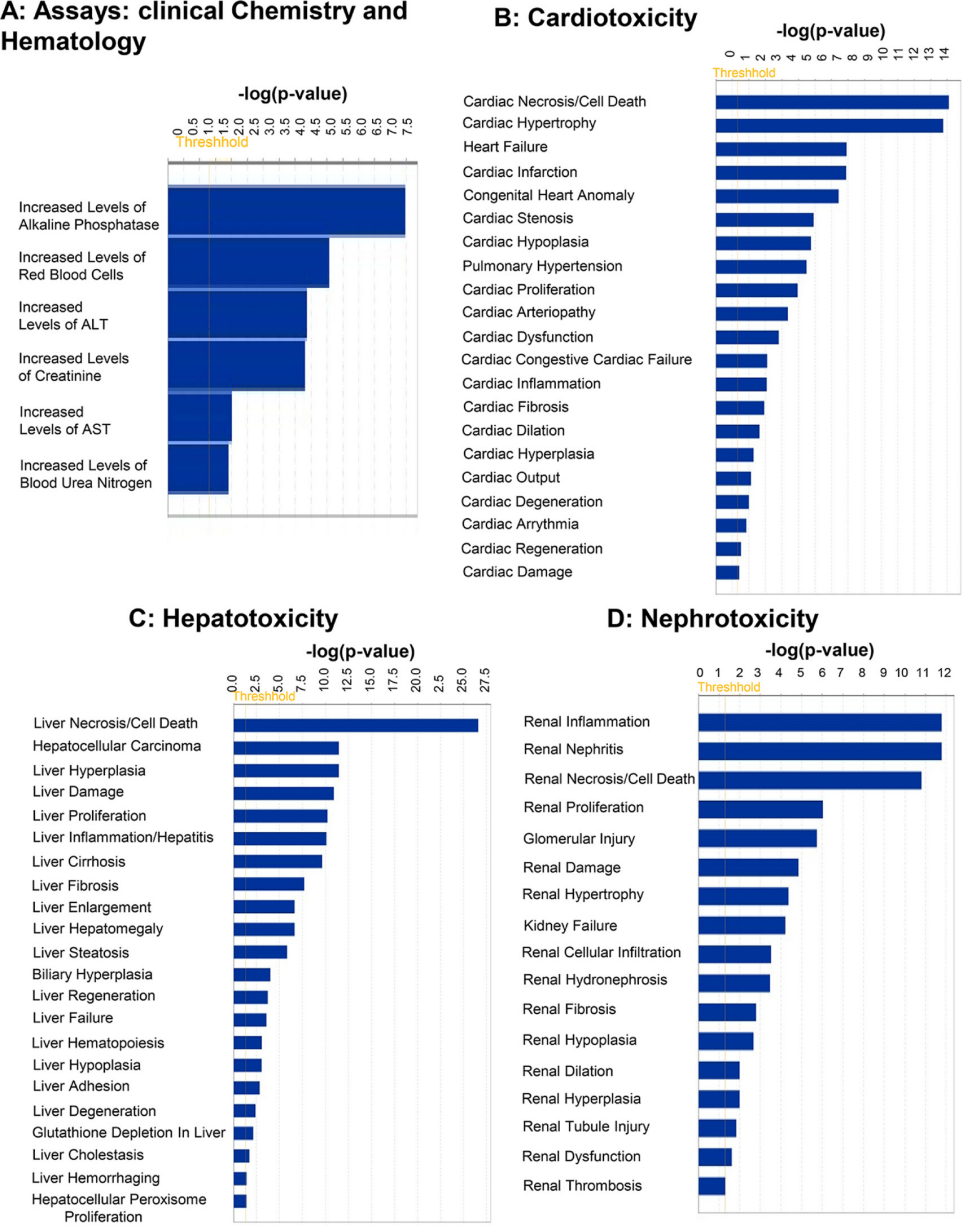
Top predicted “Tox Functions” targeted by mRNAs whose expression is regulated by miRNAs differentially loaded in Exo^Hypoxic^ from human PCa LNCaP cells Data were analyzed by IPA and top (**A**) ‘Assays: Clinical Chemistry and Hematology’; (**B**) ‘Cardiotoxicity’; (**C**) ‘Hepatotoxicity’; and (**D**) ‘Nephrotoxicity’ are presented. Fisher’s exact test was used to calculate a *P*-value determining the probability of the association between the genes and the dataset in the functions.

Among the Tox Functions mentioned above, the top five Assays were: Clinical chemistry and hematology, including increased levels of alkaline phosphatase; increased levels of red blood cells; increased levels of alanine transferase; increased levels of creatinine; and increased levels of AST (Figure 3A). The top five functions affected in cardiotoxicity includes Cardiac Necrosis/Cell Death; Cardiac Hypertrophy; Heart Failure; Cardiac Infarction; and Congenital Heart Anomaly (Figure 3B). The top five functions affected in Hepatotoxicity include Liver Necrosis/Cell Death; Hepatocellular Carcinoma; Liver Hyperplasia/Hyperproliferation; Liver Damage; and Liver Proliferation (Figure 3C). The top five functions affected in Nephrotoxicity include Renal Inflammation; Renal Nephritis; Renal Necrosis/Cell Death; Renal Proliferation; and Glomerular Injury (Figure 3D).

### Heatmap of predicted KEGG pathways

**Figure 4:**
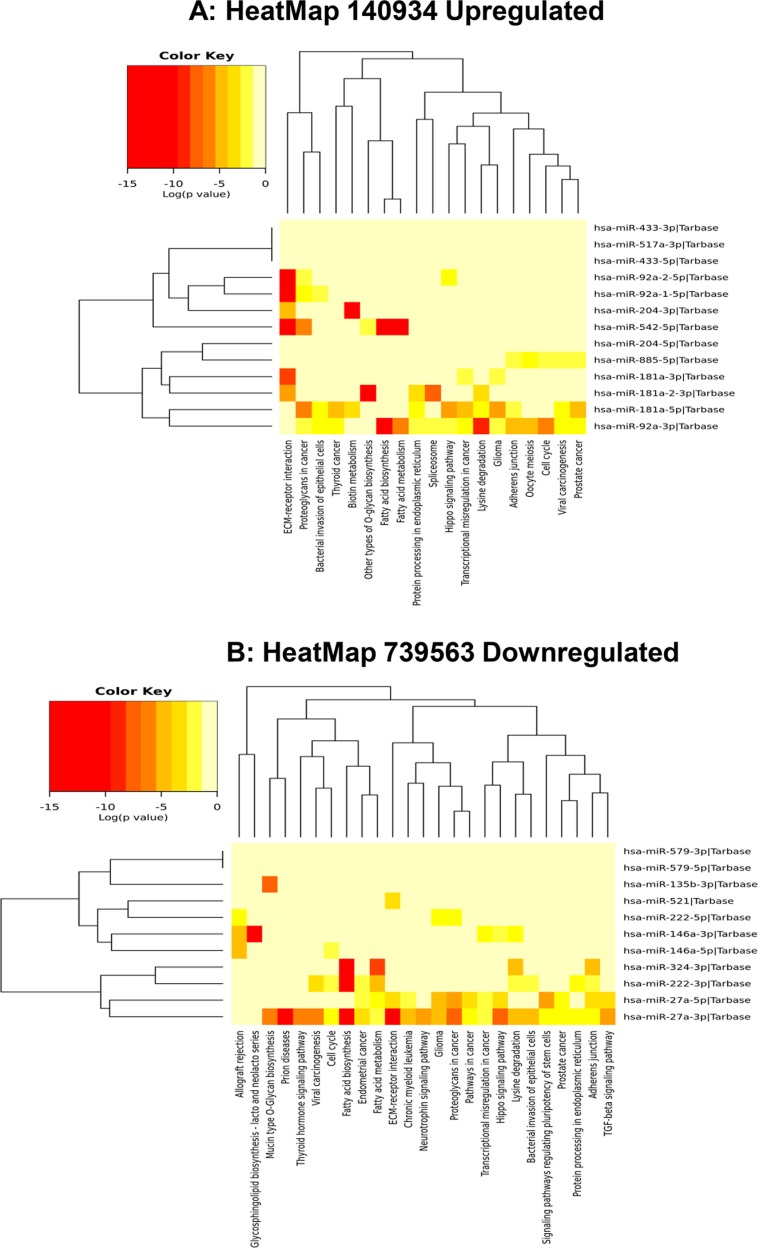
Heat map of up- and downregulated miRNAs and their predicted pathways (**A**) Heat map-predicting pathways of upregulated miRs in Exo^Hypoxic^. **(B)** Heat map-predicting pathways of downregulated miRs in Exo^Hypoxic^. Red indicates up-regulated and yellow indicates down-regulated miRs. The heatmap was generated using mirPath v.3 (Diana Tools) as described.

**Figure 5:**
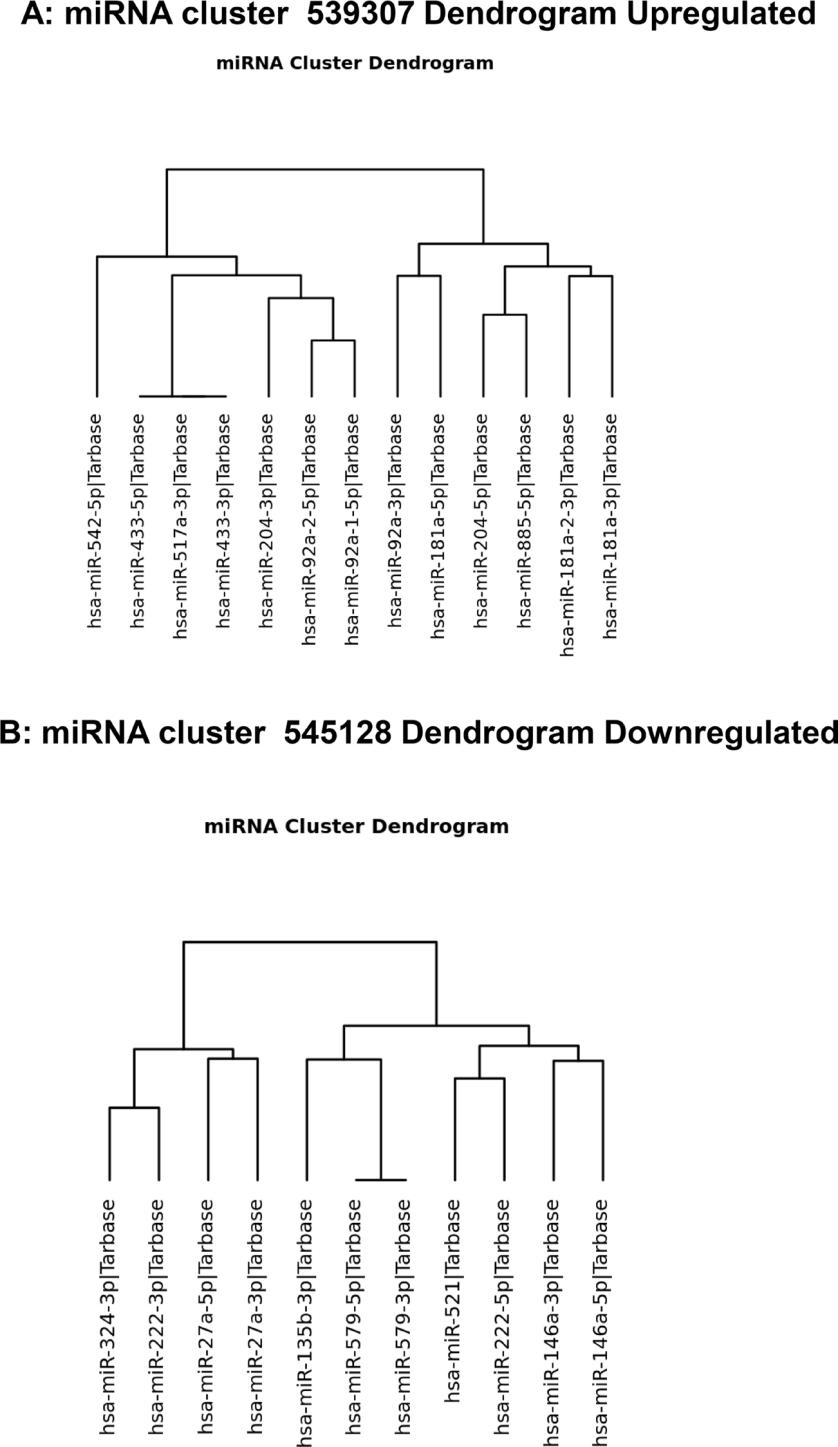
Dendrogram of hierarchical clustering up- and downregulated miRNAs (**A**) Hierarchical clustering of upregulated miRs in Exo^Hypoxic^. (**B**) Hierarchical clustering of downregulated miRs in Exo^Hypoxic^.

Using MirPath v.3 analysis of upregulated miRs (Diana Tools Software), 19 pathways such as PCa, cell cycle, and Hippo signaling pathways were identified (Figure 4A). Pathways associated with 11 downregulated miRNAs were also analyzed and the list includes TGF-beta, adherent junction, PCa, neurotrophin and regulating pluripotency of stem cells signaling pathways (Figure 4B). Differential expression of miRNAs under hypoxic conditions were associated with different biological processes that occur in PCa. Up- and down-regulated miRNAs in response to hypoxia were also analyzed by hierarchical clustering and miRNA cluster dendrograms (Figure 5A and 5B).

### Characterization of Exo^Hypoxic^ specific miRNAs in PCa patients

**Figure 6:**
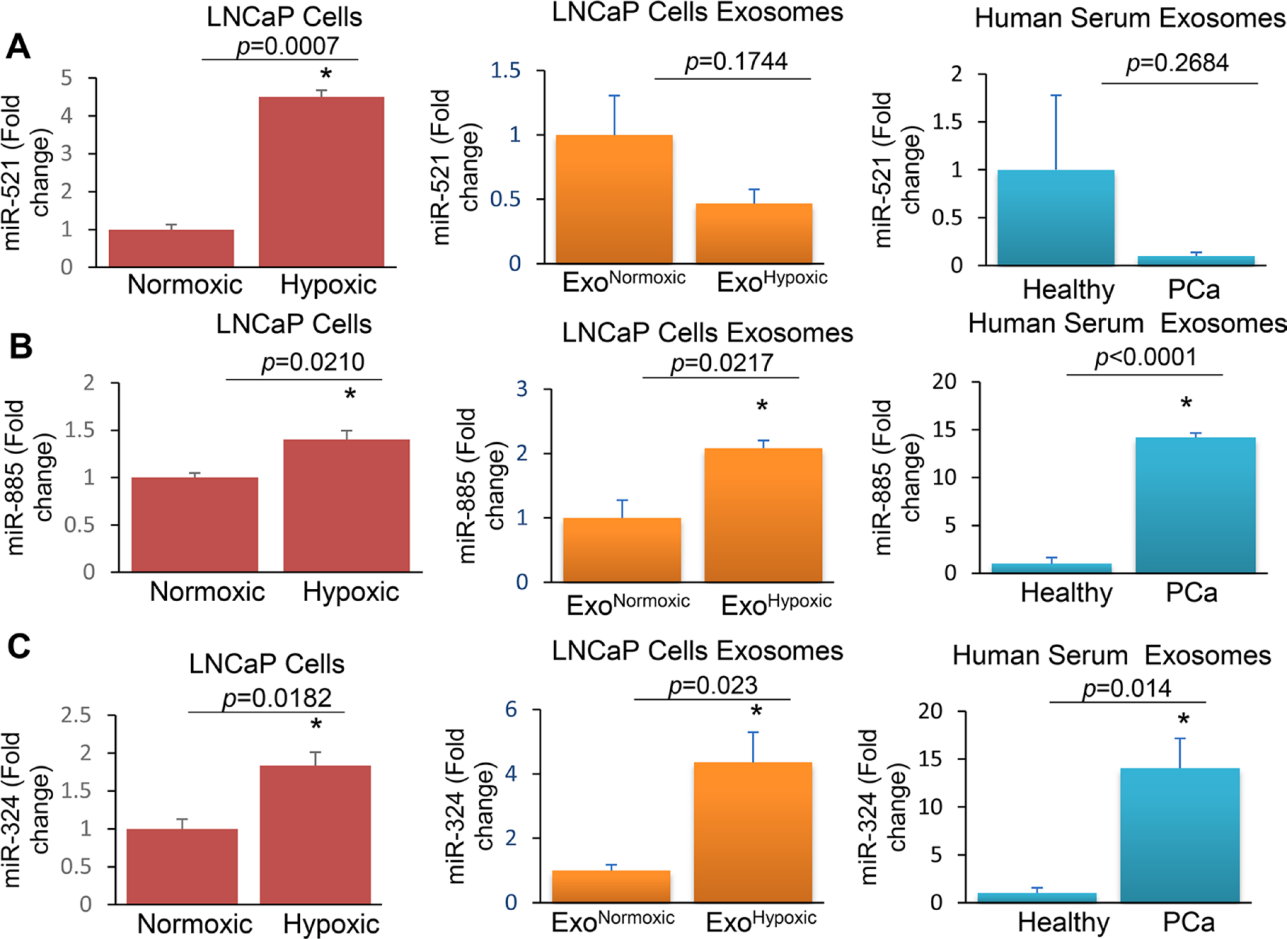
Levels of miR-521, miR-885, and miR-324 in LNCaP cells, Exo^Hypoxic^ and exosomes derived from the serum of PCa patients RNA was extracted from LNCaP cells cultured under hypoxic (1% O_2_) and normoxic (21% O_2_) conditions as well as exosomes secreted by the cells (Exo^Hypoxic^ and Exo^Normoxic^). In addition, RNA was extracted from exosomes isolated from the serum of healthy individuals and PCa patients. cDNA was synthesized and real-time PCR was performed. RNU6-2 (U6) was used as an internal control and q-PCR results were represented as a fold change relative to normoxic or healthy subjects for miR-521 (**A**), miR-885 (**B**) and miR-324 (**C**). ^*^denotes significance at *p* < 0.05 and *p* value is presented.

Next, we analyzed the levels of some LNCaP Exo^Hypoxic^ miRNAs (up- or down-regulated in the microRNA array) in exosomal samples from serum of patients with prostate cancer (patients’ characteristics are listed in Table 4). We first validated array data in LNCaP cells and Exo^Hypoxic^ by q-PCR. Similar to array results, miR-521 levels were lower in Exo^Hypoxic^ compared to Exo^Normoxic ,^ although cellular levels of miR-521 were higher in hypoxic LNCaP cells compared to normoxic LNCaP cells (Figure 6A). Importantly, lower miR-521 levels were observed in exosomes from PCa patients (*n* = 12) compared to healthy individuals (*n* = 7). Furthermore, similar to microarray data, miR-885 levels were higher in Exo^Hypoxic^ compared to Exo^Normoxic^, and a similar increase was also observed in hypoxic LNCaP cells compared to normoxic LNCaP cells (Figure 6B). Intriguingly, miR-885 levels in exosomes isolated from the serum of PCa patients were significantly higher compared to exosomes from the serum of healthy individuals (Figure 6B). q-PCR results showed that Exo^Hypoxic^ had higher levels of miR-324 compared to Exo^Normoxic^, although array data showed decreased miR-324 levels in Exo^Hypoxic^ (Figure 6C). Furthermore, miR-324 levels were higher in hypoxic LNCaP cells compared to normoxic LNCaP cells (Figure 6C). Similar to Exo^Hypoxic^, we observed higher miR-324 levels in exosomes in serum of PCa patients versus healthy individuals (Figure 6C). Overall, all three miRs analyzed in serum exosome samples showed trends similar to those in Exo^Hypoxic^.

**Table 4:** Patients’ characteristics

Parameter		Normal	PCa patients
**Age (years)**	Mean ± SD	59.9 ± 6.7	63.6 ± 8.2
**Gleason score (N)**	>7		4
	= 7		2
	<7		6
**Prostate-specific antigen (ng/ml)**	Mean ± SD		10.8 ± 6.6
**Tumor volume (mm**^3^**)**	Mean ± SD		18.45 ± 13.5
**Stage**	T2c		6
	T3a		4
	T3b		2

**Figure 7:**
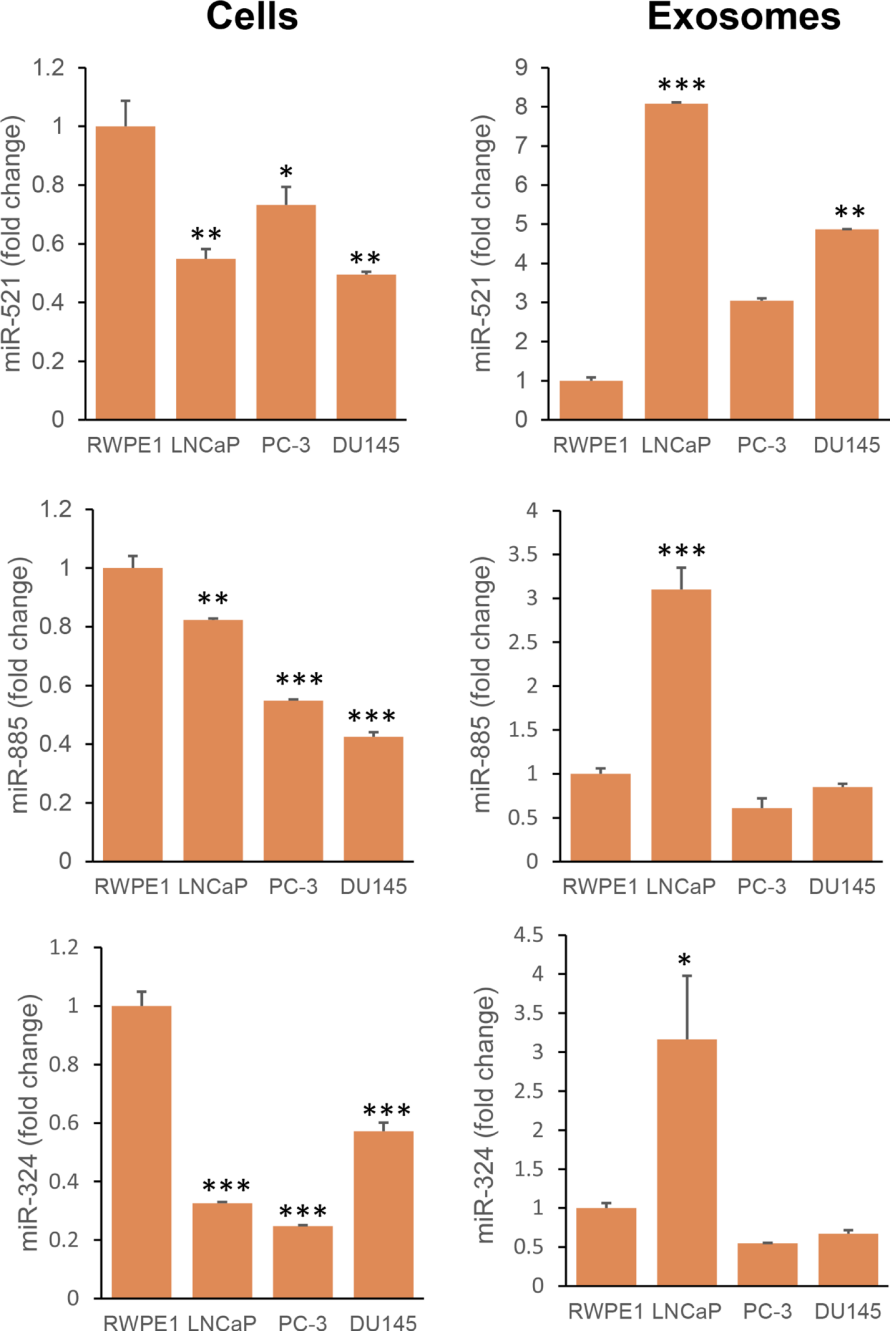
Expression of miR-521, miR-885, and miR-324 in non-neoplastic and neoplastic prostate cells and their exosomes Exosomes were isolated from the conditioned medium of RWPE1 (non-neoplastic prostate epithelial cells) and PCa cells (LNCaP, PC-3 and DU145). RNA was extracted from cells and exosomes. Real-time PCR was performed as described in the Methods section. Expression was calculated as a fold change relative to RWPE1. Data was analyzed by One way ANOVA followed by Bonferroni *t*-test. ^*^p < 0.05; ^**^p < 0.01; ^***^p < 0.001.

Next, we compared levels of the miRNAs (521, 885 and 324) among non-neoplastic prostate epithelial (RWPE1) and various prostate cancer cell lines – LNCaP (AR+), PC-3 (AR–) and DU145 (AR–) – and exosomes secreted by these cells. Expression of all three miRNAs was significantly lower in the prostate cancer cells. Further, exosomes secreted by LNCaP cells had significantly higher levels of all three miRNAs compared to the other cell lines (Figure 7).

### TCGA analysis of Exo^Hypoxic^ specific miRs

The Cancer Genome Atlas (TCGA) prostate adenocarcinoma (PRAD) genomic dataset was used to compare associations between miRs of interest and pathological measures of PCa aggressiveness in 488 primary prostate tumors. Two miRs, hsa-miR-204 (up-regulated in hypoxia) and hsa-miR-222 (down-regulated in hypoxia), displayed correlated expression patterns in prostate tumors (Pearson *R* = 0.66, *p* < 0.0001), and showed statistically significant inverse correlations with tumor T stage, N stage and Gleason score (Table 5).

**Table 5:** Associations between miR expression and pathological scores of prostate tumors

	Change under hypoxia	Pathology T stage (Pearson R, *P* val)	Pathology nodal [N] stage (Odds Ratio [CI], *P* val)	Gleason score (Pearson R, *P* val)
**hsa-miR-204**	UP	–0.15, 1.1E-03	0.73 [0.57–0.95], 1.8E-02	–0.24, 7.6E-08
**hsa-miR-222**	DOWN	–0.25, 1.5E-08	0.58 [0.42–0.79], 4.0E-04	–0.32, 5.3E-13

## DISCUSSION

Hypoxia is a hallmark of solid tumors, endowing them with malignant, aggressive, and treatment-refractory characteristics [20]. Exosomes play an important role in transferring message from hypoxic cancer cells to normoxic cells in the tumor microenvironment, thereby contributing toward cancer growth, progression, and metastasis [14, 18, 21, 22]. Clinical studies clearly suggest the potential of hypoxia/hypoxia signaling biomarkers as a tool for prognosis of the disease and making key treatment decisions [20]. However, more reliable non-invasive methods are needed for longitudinal measures of hypoxia in tumors. Exosomes could be useful in this role. Hence, in the present study we sought to characterize a hypoxic exosomal miRNA signature for PCa cells and to assess the clinical utility of such a signature.

The top miRNAs that we found to be significantly modulated in Exo^Hypoxic^ have functional relevance in various cancers. For example, Jin *et al.* recently reported that miR-517a-3p promotes lung cancer cell proliferation and invasion by targeting FOXJ3 expression [23]. This microRNA is now used to help classify hepatocellular carcinoma [24]. Similarly, miR-92 is involved in induction of angiogenesis and enhancement of endothelial cell migration [25]. The prognostic and biological significance of miR-127 expression in breast cancer was reported by Wang *et al.* [26]. Parikh *et al.* reported that in ovarian cancer miR-181a promotes TGF-β-mediated epithelial-to-mesenchymal transition via repression of its functional target, Smad7 [27].

Our data in the present study are consistent with the above findings showing the upregulation of miR-517, miR-92a, miR-127, and miR-181a in Exo^Hypoxic^. Furthermore, in a previous study of non-small cell lung cancer cells, miR-146a inhibited cell growth and cell migration but induced apoptosis [28]; we observed decreased levels of miR-146a in Exo^Hypoxic^. In another study, Tadorova *et al.* showed that miR-204 is dysregulated in metastatic PCa *in vitro* [29]. In colon cancer, dysregulation of miR-324-5p resulted in macrophage dysfunction [30]. A later study highlighted the role of miR-324-5p in suppression of hepatocellular carcinoma by counteracting extracellular matrix degradation [31].

Similarly, Heyn *et al.* described the importance of miR-335 in breast cancer development, since it targets the BRCA1 regulatory cascade [32]. Gong *et al.* reported that miR-335 inhibits small cell lung cancer bone metastasis via IGF-IR and RANKL pathways [33]. Another study identified miR-335 as an independent prognostic marker in epithelial ovarian cancer [34]. A recent study on miR-502 reported its potential tumor suppressor role in colon cancer [35]; in the present study, this miR was downregulated in Exo^Hypoxic^. Taken together, the literature suggests that significantly modulated miRNAs in Exo^Hypoxic^ are relevant in PCa, and should be studied further for their usefulness as a molecular biomarker for hypoxic tumors and to assess their targets.

In the present study, we observed a similar expression trend in Exo^Hypoxic^ (compared to Exo^Normoxic^) and exosomes from PCa patients’ serum (compared to healthy individuals) for all 3 miRs (521, 885 and 324). Arrays and q-PCR analyses showed that miR-521 levels are lower in Exo^Hypoxic;^ however, miR-521 levels were higher in hypoxic LNCaP cells, suggesting a differential loading of miRs in exosomes. We anticipate that the differential release of miRNAs via exosomes, in the conditioned medium or blood stream, is one way cells can communicate with each other to create specific biological effects [36]. Furthermore, arrays showed that miR-324 levels are decreased in Exo^Hypoxic^; however, q-PCR results showed an increase in miR-324 in Exo^Hypoxic^. These results highlight that validation of array data by q-PCR is important in identifying novel miRNAs as possible biomarkers. Further, there remain different pitfalls of using each technique [37, 38]. Biological and non-biological procedures mostly affect microarray and q-PCR results [39]. Despite these limitations, it is encouraging to see similar trends in miRNA levels in Exo^Hypoxic^ and exosomes from PCa patients.

In the present study, IL-6 signalling was the top hit in canonical pathways. This signaling pathway has been implicated in several human cancers [40, 41]. It is a multifunctional pro-inflammatory cytokine expressed in specimens from patients with PCa and in multiple cell lines [42]. Earlier studies revealed that IL-6 mediates resistance to chemotherapy in PCa [43]. Bao *et al.* reported that IL6 was involved in hypoxia-induced aggressiveness in PCa PC-3 cells [16]. Jeong *et al.* showed that IL-6 production is linked to hypoxia-induced activation of MAP kinase, HIF-1α, and NF-κB [44]. Therefore, it is plausible that miRNA/s loaded in Exo^Hypoxic^ could affect the mRNA expression of genes that regulate IL6 expression in target cells, a question that merits further explortion.

Earlier, we reported that Exo^Hypoxic^ are associated with remodelling of the epithelial adherens junction and cytoskeleton signaling [18]. In the present study, IPA analyses identified ‘Cellular Movement’ as the top hit under Molecular and cellular functions, which is consistent with our earlier finding. However, the specific pathways and key molecules involved in this biological response are still not clear. For example, we reported that Exo^Hypoxic^ treatment promoted invasiveness in PC-3 cells *via* decreasing E-cadherin expression and increasing nuclear β-catenin expression [18]. Parikh *et al.* have shown that miR-181a targets E-cadherin expression in ovarian cancer cells [27]. In the present study, we observed an increase in miR-181a (1.5 fold) in Exo^Hypoxic^ compared to Exo^Normoxic^. Further, the molecular effects of miRNAs loaded in Exo^Hypoxic^ on local and distant cell types in the tumor microenvironment remain unclear.

Our IPA analysis identified ‘cardiotoxicity pathway’ as a target pathway, but in the literature this has not been consistently reported. In the Rotterdam randomized screening trial [45], PCa treatment did not increase the risk of dying as a result of cardiovascular causes. However, in another study, androgen-deprivation therapy was associated with a 5% absolute excess risk of cardiac-specific mortality at 5 years in men with congestive heart failure or past myocardial infarction [46].

Overall, results from the present study showed significantly different levels of key miRNAs in Exo^Hypoxic^ compared with Exo^Normoxic^. These outcomes correlate well in PCa patients’ samples, suggesting the utility of exosomes as possible biomarkers for hypoxia levels in tumors. Furthermore, our results support our previous findings on hypoxia-induced biological effects in PCa. In the future, we will further characterize the biological effects of key miRNAs in Exo^Hypoxic^ on components of the local and distant tumor microenvironment, and mechanisms involved in their uptake by target cells.

## MATERIALS AND METHODS

### Cell lines and reagents

Human LNCaP, PC-3, and DU145 PCa cell lines and non-neoplastic prostate epithelial RWPE-1 cells were purchased from ATCC (Manassas, VA). miRNeasy micro kits were obtained from Qiagen (Valencia, CA). TaqMan^®^ Array MicroRNA Cards with pre-amplification and TaqMan^®^ MicroRNA Reverse Transcription Kits were purchased from Life Technologies, Applied Biosystems (Foster City, CA). RPMI-1640 medium and other cell culture materials were from Invitrogen Corporation (Gaithersburg, MD). All other reagents were obtained in the highest purity that is commercially available.

### Cell culture and hypoxia exposure

PC-3, DU145, and LNCaP cells were cultured in a humidified environment at 37° C and 5% CO_2_ as adherent monolayer in DMEM or RPMI1640 (for LNCaP) medium supplemented with 10% fetal bovine serum (FBS) in presence of 100 U/mL penicillin G and 100mg/mL streptomycin sulfate. RWPE-1 cells were grown in keratinocyte serum-free media supplemented with bovine pituitary extract and epidermal growth factor (Life Tech. Corp., Grand Island, NY). Hypoxia experiments were performed in a hypoxia chamber at 1% O_2_ at 37° C in a 5% CO_2_ humidified environment.

### Exosome collection and miRNA isolation

Exosomes were collected from the conditioned media of LNCaP cells cultured under normoxic or hypoxic conditions for 72 h by ultracentrifugation as we previously described [18, 19]. Exo^Hypoxic^ and Exo^Normoxic^ miRNA was isolated using the miRNeasy micro kit (Qiagen) following the vendor’s instructions. Briefly, exosomes were disrupted and homogenized in RLT buffer (Qiagen), and the rest of the procedure was performed according to the manufacturer’s protocol. RNA concentrations and purity were confirmed by the spectrophotometric ratio using absorbance measurements at wavelengths of 260 nm and 280 nm on a Nanodrop 2000 (Thermo Fisher, Wilmington, DE).

### miRNA expression profile

miRNA in Exo^Hypoxic^ and Exo^Normoxic^ were analyzed using TaqMan^®^ Array MicroRNA Cards with pre-amplification kit (Life Technologies, Applied Biosystems). 350 ng of miRNA was used as input in each reverse transcription (RT) reaction. The RT reaction and pre-amplification steps were done according to the manufacturer’s instructions. miRNAs were reverse transcribed using TaqMan^®^ MicroRNA Reverse Transcription Kit (Life Technologies, Applied Biosystems). RT reaction products from the exosome sample were further amplified using the Custom PreAmp Primer Pool. The expression profile of miRNAs was determined using the TaqMan^®^ Universal Master Mix II (Life Technologies, Applied Biosystems) in an Applied Biosystems 7900HT thermal cycler using the manufacturer’s recommended program. Finally, all the raw data from each array were retrieved from the 7900HT and run on Data Assist Software ver.3.1 (Applied Biosystems). In another set of experiments, we collected exosomes from the conditioned medium of RWPE1, LNCaP, PC-3 and DU145 cells; RNA was isolated from cells and exosomes, followed by miRNA cDNA synthesis (Applied Biological Materials Inc., BC, Canada). Real-time PCR was performed using SYBR Green master mix on a Bio-Rad CFX96 detection system (Bio-Rad, Hercules, CA). The fold change in expression levels was determined using RNU6 and 5S rRNA as an internal control.

### miRNA target prediction and pathway analysis

miRNA targets prediction was carried out using the microRNA Target Filter function in Ingenuity Pathways Analysis software (IPA, Ingenuity Systems, Redwood City, CA). IPA’s microRNA Target Filter employs experimentally validated and predicted mRNA targets from TargetScan, TarBase, miRecords, and the Ingenuity^®^ Knowledge Base. Subsequently, the predicted target mRNAs were subjected to pathway exploration by core analysis in IPA considering only the relationships where the confidence limit was “experimentally observed”. The heatmap and clustering of predicted KEEG pathways of up- and downregulated miRs in Exo^Hypoxic^ were performed according to Vlachos *et al.* [47].

### Validation of array data and detection of exosome-associated miRNAs in serum from patients with PCa

Serum was collected from 12 patients with PCa (clinical information summarized in Table 4) and 7 healthy individuals following a protocol approved by the IRB from Texas A&M Health Science Center. MiRCURY™ exosome isolation kits were used according to the manufacturer’s directions (Exiqon). Briefly, 0.6 ml serum was centrifuged at 10,000 × g to remove cell debris and 200 µl of exosome precipitation solution was added to the clear supernatant and incubated for 60 minutes at 4° C. Samples were centrifuged at 1,500 *g* for 30 min and the exosomal pellet was re-suspended in 300 µl of resuspension buffer for subsequent analyses. Total RNA was extracted using modified protocol from miRCURY™ RNA Isolation Kits-Biofluids (Exiqon). Briefly, to 300 µl of re-suspended exosomes, 90 µl of lysis solution was added to samples and the protocol for RNA extraction was completed. For mature miRNA cDNA synthesis, miScript II RT kit (Qiagen) was used for q-PCR amplification according to the manufacturer’s instructions. Quantitative real-time PCR was performed using a Bio-Rad CFX96 iCycler device. U6 was used as a reference gene for normalizations and the relative amount of each miRNA was calculated as ΔCt = Ct miRNA-Ct U6. The fold change of each miRNA was calculated using the comparative Ct method (ΔΔCt), where mean ΔCt of control samples for the miRNA subtracted from that of each sample. The fold change (FC) calculated according to this formula FC = 2^−ΔΔCt^. Each sample was used in triplicate and repeated at least twice.

### TCGA analysis

Level 3-processed miR-Seq profiles and clinical data files from the TCGA PRAD dataset were downloaded from the Broad Institute’s FireBrowse (Release 1.1.35) website (http://firebrowse.org/). Of the 494 tumors sequenced, 488 were associated with corresponding clinical data related to pathological T stage, nodal status (N stage) and Gleason score. Associations between miR log2 RPKM expression values and pathological variables were assessed by Pearson correlation (T stage, Gleason score) and multiple logistic regression analysis (N stage).

### Statistical analyses

Significant differences between levels of miRNA in normoxic and hypoxic conditions were determined using Student’s *t*-tests. All miRNA results from qPCR analysis were presented as fold change relative to healthy individuals or RWPE1 cells using RNU6 and 5S rRNA as internal controls. Statistical analyses were performed using a Mann-Whitney test (GraphPad Software, Inc., La Jolla, CA). A *p*-value of less than 0.05 was considered significant. For data analysed in IPA, Fisher’s exact test was used to calculate a *p*-value determining the probability of the association between the genes and the dataset in the functions or the pathways.

## SUPPLEMENTARY MATERIALS TABLE





## Abbreviations

ADT: Androgen-deprivation therapy
CAF: Cancer-associated fibroblasts
CSM: Cardiac-specific mortality
Exo^Hypoxic^: Exosomes secreted by cells under hypoxic condition
Exo^Normoxic^: Exosomes secreted by cells under normoxic condition
FBS: Fetal bovine serum
IPA: Ingenuity pathways analysis
KEGG: Kyoto encyclopedia of genes and genomes
miRNA: microRNA
PCa: Prostate cancer
PRAD: Prostate adenocarcinoma
PSA: Prostate-specific antigen
q-PCR: Quantitative-polymerase chain reaction
TCGA: The Cancer Genome Atlas
