# Supplementary material for: Exosomal microRNA profiling to identify hypoxia-related biomarkers in prostate cancer
Source: Oncotarget. 2018 Feb 17;9(17):13894–910. doi: 10.18632/oncotarget.24532 (PMC5862624; doi:10.18632/oncotarget.24532)
Supplement: Supplementary file 1 [file oncotarget-09-13894-s001.pdf]

Exosomal microRNA profiling to identify hypoxia-related biomarkers in prostate cancer

SUPPLEMENTARY MATERIALS

Supplementary Table 1: List of miRNA expressed in Exo^{Normoxic} and Exo^{Hypoxic}. See Supplementary_Table_1
