# Supplementary material for: Exosomal microRNA profiling to identify hypoxia-related biomarkers in prostate cancer
Source: Oncotarget. 2018 Feb 17;9(17):13894–910. doi: 10.18632/oncotarget.24532 (PMC5862624; doi:10.18632/oncotarget.24532)
Supplement: Supplementary file 2 [file oncotarget-09-13894-s002.docx]

**Supplementary Table 1:** List of miRNA expressed in Exo^Normoxic^ and Exo^Hypoxic^.

| Sl No | miRNA_Common_ |
| --- | --- |
| 1 | hsa-miR-28-3p-002446 |
| 2 | hsa-miR-521-001122 |
| 3 | hsa-miR-425-5p-001516 |
| 4 | hsa-miR-374-000563 |
| 5 | hsa-miR-451-001141 |
| 6 | hsa-miR-200c-002300 |
| 7 | hsa-miR-27a-000408 |
| 8 | hsa-miR-324-3p-002161 |
| 9 | hsa-miR-517a-002402 |
| 10 | hsa-miR-885-5p-002296 |
| 11 | hsa-miR-410-001274 |
| 12 | hsa-miR-143-002249 |
| 13 | hsa-miR-92a-000431 |
| 14 | hsa-miR-335-000546 |
| 15 | hsa-miR-579-002398 |
| 16 | hsa-miR-502-001109 |
| 17 | hsa-miR-181a-000480 |
| 18 | hsa-miR-222-002276 |
| 19 | hsa-miR-127-000452 |
| 20 | hsa-miR-542-5p-002240 |
| 21 | hsa-miR-301-000528 |
| 22 | hsa-miR-135b-002261 |
| 23 | hsa-miR-146a-000468 |
| 24 | hsa-miR-433-001028 |
| Sl No | miRNA_Common_ |
| 25 | hsa-miR-491-001630 |
| 26 | hsa-miR-204-000508 |
| 27 | hsa-miR-223-002295 |
| 28 | hsa-miR-146b-001097 |
| 29 | hsa-miR-519e-002370 |
| 30 | hsa-miR-188-3p-002106 |
| 31 | hsa-miR-221-000524 |
| 32 | hsa-miR-139-5p-002289 |
| 33 | hsa-miR-598-001988 |
| 34 | hsa-miR-376c-002122 |
| 35 | hsa-miR-299-5p-000600 |
| 36 | hsa-miR-876-3p-002225 |
| 37 | hsa-miR-149-002255 |
| 38 | hsa-miR-26b-000407 |
| 39 | RNU48-001006 |
| 40 | hsa-miR-32-002109 |
| 41 | hsa-miR-489-002358 |
| 42 | hsa-miR-212-000515 |
| 43 | hsa-miR-484-001821 |
| 44 | hsa-miR-570-002347 |
| 45 | hsa-miR-330-000544 |
| 46 | hsa-miR-22-000398 |
| 47 | hsa-miR-19b-000396 |
| 48 | hsa-miR-652-002352 |
| Sl No | miRNA_Common_ |
| 49 | hsa-miR-20a-000580 |
| 50 | hsa-miR-9-000583 |
| 51 | hsa-miR-301b-002392 |
| 52 | hsa-miR-494-002365 |
| 53 | U6 snRNA-001973 |
| 54 | U6 snRNA-001973 |
| 55 | U6 snRNA-001973 |
| 56 | U6 snRNA-001973 |
| 57 | hsa-miR-376a-000565 |
| 58 | hsa-miR-26a-000405 |
| 59 | hsa-miR-744-002324 |
| 60 | mmu-miR-140-001187 |
| 61 | hsa-miR-31-002279 |
| 62 | hsa-miR-500-002428 |
| 63 | hsa-miR-509-5p-002235 |
| 64 | hsa-miR-486-001278 |
| 65 | hsa-miR-145-002278 |
| 66 | hsa-miR-518e-002395 |
| 67 | hsa-miR-362-3p-002117 |
| 68 | hsa-miR-454-002323 |
| 69 | hsa-miR-342-3p-002260 |
| 70 | hsa-miR-487b-001285 |
| 71 | hsa-miR-548a-001538 |
| 72 | hsa-miR-220-000523 |
| Sl No | miRNA_Common_ |
| 73 | hsa-miR-130a-000454 |
| 74 | hsa-miR-449b-001608 |
| 75 | hsa-miR-574-3p-002349 |
| 76 | hsa-miR-886-5p-002193 |
| 77 | hsa-miR-200a-000502 |
| 78 | hsa-miR-618-001593 |
| 79 | mmu-miR-615-001960 |
| 80 | hsa-miR-193a-3p-002250 |
| 81 | hsa-miR-15b-000390 |
| 82 | hsa-miR-597-001551 |
| 83 | hsa-miR-532-3p-002355 |
| 84 | hsa-miR-629-002436 |
| 85 | hsa-miR-224-002099 |
| 86 | hsa-miR-625-002431 |
| 87 | hsa-miR-150-000473 |
| 88 | hsa-miR-548b-001541 |
| 89 | hsa-miR-324-5p-000539 |
| 90 | hsa-miR-24-000402 |
| 91 | hsa-miR-125a-3p-002199 |
| 92 | hsa-miR-328-000543 |
| 93 | hsa-miR-142-3p-000464 |
| 94 | hsa-miR-193b-002367 |
| 95 | hsa-miR-96-000186 |
| 96 | hsa-miR-379-001138 |
| 97 | hsa-miR-183-002269 |
| 98 | hsa-miR-329-001101 |
| 99 | hsa-miR-320-002277 |
| 100 | hsa-miR-340-002258 |
| Sl No | miRNA_Common_ |
| 101 | hsa-miR-495-001663 |
| 102 | hsa-miR-34a-000426 |
| 103 | hsa-miR-627-001560 |
| 104 | hsa-miR-106b-000442 |
| 105 | hsa-miR-450a-002303 |
| 106 | hsa-let-7c-000379 |
| 107 | hsa-miR-33b-002085 |
| 108 | hsa-miR-128a-002216 |
| 109 | hsa-miR-517c-001153 |
| 110 | hsa-miR-636-002088 |
| 111 | hsa-miR-660-001515 |
| 112 | hsa-miR-197-000497 |
| 113 | hsa-miR-339-5p-002257 |
| 114 | hsa-miR-361-000554 |
| 115 | hsa-miR-103-000439 |
| 116 | hsa-miR-130b-000456 |
| 117 | hsa-let-7g-002282 |
| 118 | hsa-miR-424-000604 |
| 119 | hsa-miR-191-002299 |
| 120 | hsa-miR-576-5p-002350 |
| 121 | hsa-miR-95-000433 |
| 122 | hsa-miR-27b-000409 |
| 123 | hsa-miR-135a-000460 |
| 124 | hsa-miR-140-3p-002234 |
| 125 | hsa-miR-142-5p-002248 |
| 126 | hsa-miR-18b-002217 |
| 127 | hsa-miR-137-001129 |
| 128 | hsa-miR-369-3p-000557 |
| Sl No | miRNA_Common_ |
| 129 | hsa-miR-487a-001279 |
| 130 | hsa-miR-302a-000529 |
| 131 | hsa-miR-29a-002112 |
| 132 | hsa-miR-184-000485 |
| 133 | hsa-miR-200b-002251 |
| 134 | hsa-miR-195-000494 |
| 135 | hsa-miR-214-002306 |
| 136 | hsa-miR-380-3p-000569 |
| 137 | hsa-miR-886-3p-002194 |
| 138 | hsa-miR-367-000555 |
| 139 | hsa-miR-590-5p-001984 |
| 140 | hsa-miR-208b-002290 |
| 141 | hsa-miR-370-002275 |
| 142 | hsa-miR-29c-000587 |
| 143 | hsa-miR-655-001612 |
| 144 | RNU44-001094 |
| 145 | hsa-miR-323-3p-002227 |
| 146 | hsa-miR-615-5p-002353 |
| 147 | hsa-miR-34c-000428 |
| 148 | hsa-miR-99a-000435 |
| 149 | hsa-miR-582-3p-002399 |
| 150 | hsa-miR-296-000527 |
| 151 | hsa-miR-133a-002246 |
| 152 | hsa-miR-148a-000470 |
| 153 | hsa-miR-544-002265 |
| 154 | hsa-miR-496-001953 |
| 155 | hsa-miR-152-000475 |
| 156 | hsa-miR-486-3p-002093 |
| Sl No | miRNA_Common_ |
| 157 | hsa-miR-365-001020 |
| 158 | hsa-miR-520f-001120 |
| 159 | hsa-miR-485-3p-001277 |
| 160 | hsa-miR-25-000403 |
| 161 | hsa-miR-891a-002191 |
| 162 | hsa-miR-551b-001535 |
| 163 | hsa-miR-422a-002297 |
| 164 | hsa-miR-10a-000387 |
| 165 | hsa-miR-199a-3p-002304 |
| 166 | hsa-miR-186-002285 |
| 167 | hsa-miR-20b-001014 |
| 168 | hsa-miR-30b-000602 |
| 169 | hsa-miR-493-002364 |
| 170 | hsa-miR-616-002414 |
| 171 | hsa-miR-185-002271 |
| 172 | hsa-miR-302b-000531 |
| 173 | hsa-miR-429-001024 |
| 174 | hsa-miR-105-002167 |
| 175 | hsa-miR-532-001518 |
| 176 | hsa-miR-377-000566 |
| 177 | hsa-let-7a-000377 |
| 178 | hsa-miR-411-001610 |
| 179 | hsa-miR-139-3p-002313 |
| 180 | hsa-let-7b-002619 |
| 181 | hsa-miR-561-001528 |
| 182 | hsa-miR-19a-000395 |
| 183 | hsa-miR-133b-002247 |
| 184 | hsa-miR-382-000572 |
| Sl No | miRNA_Common_ |
| 185 | hsa-miR-202-002363 |
| 186 | hsa-let-7f-000382 |
| 187 | hsa-miR-125b-000449 |
| 188 | hsa-miR-450b-3p-002208 |
| 189 | hsa-miR-122-002245 |
| 190 | hsa-miR-449-001030 |
| 191 | hsa-miR-147-000469 |
| 192 | hsa-miR-219-000522 |
| 193 | hsa-miR-23a-000399 |
| 194 | hsa-miR-218-000521 |
| 195 | hsa-miR-452-002329 |
| 196 | hsa-miR-345-002186 |
| 197 | hsa-miR-215-000518 |
| 198 | ath-miR159a-000338 |
| 199 | hsa-miR-98-000577 |
| 200 | hsa-let-7e-002406 |
| 201 | hsa-miR-628-5p-002433 |
| 202 | hsa-miR-545-002267 |
| 203 | hsa-miR-210-000512 |
| 204 | hsa-miR-337-5p-002156 |
| 205 | hsa-miR-520b-001116 |
| 206 | hsa-miR-502-3p-002083 |
| 207 | hsa-miR-331-5p-002233 |
| 208 | hsa-miR-539-001286 |
| 209 | hsa-miR-182-002334 |
| 210 | hsa-miR-18a-002422 |
| 211 | hsa-let-7d-002283 |
| 212 | hsa-miR-29b-000413 |
| Sl No | miRNA_Common_ |
| 213 | hsa-miR-518b-001156 |
| 214 | hsa-miR-374-5p-001319 |
| 215 | hsa-miR-373-000561 |
| 216 | hsa-miR-219-1-3p-002095 |
| 217 | hsa-miR-205-000509 |
| 218 | hsa-miR-211-000514 |
| 219 | hsa-miR-193a-5p-002281 |
| 220 | hsa-miR-132-000457 |
| 221 | hsa-miR-518d-001159 |
| 222 | hsa-miR-671-3p-002322 |
| 223 | hsa-miR-576-3p-002351 |
| 224 | hsa-miR-548d-001605 |
| 225 | hsa-miR-375-000564 |
| 226 | hsa-miR-346-000553 |
| 227 | hsa-miR-141-000463 |
| 228 | hsa-miR-331-000545 |
| 229 | hsa-miR-501-3p-002435 |
| 230 | hsa-miR-100-000437 |
| 231 | hsa-miR-15a-000389 |
| 232 | hsa-miR-99b-000436 |
| 233 | hsa-miR-138-002284 |
| 234 | hsa-miR-93-001090 |
| 235 | hsa-miR-124a-001182 |
| 236 | hsa-miR-363-001271 |
| 237 | hsa-miR-302c-000533 |
| 238 | hsa-miR-30c-000419 |
| 239 | hsa-miR-126-002228 |
| 240 | hsa-miR-499-3p-002427 |
| Sl No | miRNA_Common_ |
| 241 | hsa-miR-455-3p-002244 |
| 242 | hsa-miR-523-002386 |
| 243 | hsa-miR-372-000560 |
| 244 | hsa-miR-134-001186 |
| 245 | hsa-miR-362-001273 |
| 246 | hsa-miR-548a-5p-002412 |
| 247 | hsa-miR-517b-001152 |
| 248 | hsa-miR-208-000511 |
| 249 | hsa-miR-490-001037 |
| 250 | hsa-miR-125a-5p-002198 |
| 251 | hsa-miR-708-002341 |
| 252 | hsa-miR-28-000411 |
| 253 | hsa-miR-409-5p-002331 |
| 254 | hsa-miR-101-002253 |
| 255 | hsa-miR-17-002308 |
| 256 | hsa-miR-483-5p-002338 |
| 257 | hsa-miR-196b-002215 |
| 258 | hsa-miR-423-5p-002340 |
| Sl No | miRNA_Common_ |
| 259 | hsa-miR-519a-002415 |
| 260 | hsa-miR-153-001191 |
| 261 | hsa-miR-875-3p-002204 |
| 262 | hsa-miR-192-000491 |
| 263 | hsa-miR-16-000391 |
| 264 | hsa-miR-642-001592 |
| 265 | hsa-miR-129-3p-001184 |
| 266 | hsa-miR-548b-5p-002408 |
| 267 | hsa-miR-21-000397 |
| 268 | hsa-miR-155-002623 |
| 269 | hsa-miR-872-002264 |
| 270 | hsa-miR-107-000443 |
| 271 | hsa-miR-518f-002388 |
| 272 | hsa-miR-148b-000471 |
| 273 | hsa-miR-203-000507 |
| 274 | hsa-miR-509-3-5p-002155 |
| 275 | hsa-miR-505-002089 |
| 276 | hsa-miR-146b-3p-002361 |
| Sl No | miRNA_Common_ |
| 277 | hsa-miR-136-000592 |
| 278 | hsa-miR-106a-002169 |
| 279 | hsa-miR-501-001047 |
| 280 | hsa-miR-194-000493 |
| 281 | hsa-miR-758-001990 |
| 282 | hsa-miR-520e-001119 |
| 283 | hsa-miR-23b-000400 |
| 284 | hsa-miR-381-000571 |
| 285 | hsa-miR-199b-000500 |
| 286 | hsa-miR-542-3p-001284 |
| 287 | hsa-miR-455-001280 |
| 288 | hsa-miR-181c-000482 |
| 289 | hsa-miR-339-3p-002184 |
| 290 | hsa-miR-512-3p-001823 |
| 291 | hsa-miR-199a-000498 |
| 292 | hsa-miR-548c-001590 |
|  |  |
|  |  |
